# Risk perception, community myth, and practices towards COVID-19 pandemic in Southeast Ethiopia: Community based crossectional study

**DOI:** 10.1371/journal.pone.0275331

**Published:** 2022-10-03

**Authors:** Ahmednur Adem Aliyi, Musa Kumbi Ketaro, Zinash Teferu Engida, Ayele Mamo Argaw, Abduljewad Hussen Muhammed, Mesud Mohammed Hassen, Abdushekur Mohammed Abduletif, Damtow Solomon Shiferaw, Abate Lette Wodera, Sintayehu Hailu Ayene, Jeylan Kassim Esmael, Edao Sinba Etu

**Affiliations:** 1 Department of Public Health, School of Health Science, Goba Referral Hospital, Madda Walabu University, Goba, Ethiopia; 2 Department of Pharmacy, School of Health Science, Goba Referral Hospital, Madda Walabu University, Goba, Ethiopia; 3 Department of Nursing, School of Health Science, Goba Referral Hospital, Madda Walabu University, Goba, Ethiopia; 4 Department of Anatomy, School of Health Science, Goba Referral Hospital, Madda Walabu University, Goba, Ethiopia; University of Oxford, UNITED KINGDOM

## Abstract

**Objective:**

The objective of this study was to assess risk perception, community myths, and preventive practice towards COVID-19 among community in Southeast Ethiopia, 2020.

**Methods:**

Community-based cross-sectional study was conducted among 854 participants selected using a multistage sampling technique. Data were collected using a structured questionnaire adapted from previous literature. Descriptive statistics were done to summarize the variables. A generalized linear model with binary logistic specification was used to identify factors associated with risk perception and practice. Accordingly adjusted odds ratios with 95% confidence intervals were calculated and those with p-value < 0.05 were considered as significant factors associated with risk perception and practice. Cluster analysis using a linear mixed model was performed to identify factors associated with community myth and those with p-value <0.05 were reported as significant factors associated with community myth.

**Results:**

All 854 respondents gave their answer yielding 100% response rate. Of these 547 (64.1%) were male, 611 (71.5%) were rural residents, 534 (62.5%) got information about COVID-19 from TV/radio, 591 (69.2%) of them live near health facility, 265 (30.8%) have a history of substance use and 100 (11.7%) have a history of chronic illness, and 415 (48.6%) of them have a high-risk perception, 428 (50.1%) have a wrong myth about COVID-19 and 366 (42.9%) have poor practice respectively. Residence, distances from health facility and myths were significantly associated with risk perception. Occupation, knowledge, and practice were significantly associated with community myths. Also level of education, living near health facilities, having good knowledge and wrong myth were significantly associated with the practice of utilizing COVID-19 preventive respectively.

**Conclusion:**

The study found high-risk perception, high wrong community myth, and relatively low utilization of available practices towards COVID-19 and factors associated with them.

## Introduction

COVID-19 was initially started in December 2019, and later it was stated as pandemic and has been declared as a Public Health Emergency of International Concern by the WHO [1]. Approximately, 20.0% of COVID-19 patients developed severe symptoms, which included respiratory and bleeding disorders [2]. The virus can also be transmitted through the respiratory tract of patients with signs and symptoms but can also transmit from asymptomatic individuals before the onset of clinical features [3]. Susceptibility to COVID-19 looks to be associated with sociodemographic characteristics such as low education, age, and low access to information as well as with underlining co-morbidities like diabetic mellitus, cancer, and chronic respiratory illnesses [4].

The contagious COVID-19 virus outbreaks needs a urgent response from all stakeholders and communities [5]. Increasing public awareness and working in collaboration with the communities has endless benefits in curbing this pandemic [6]. People’s risk perception of the pandemic affects the utilization of available preventive measures [7]. The true risk from the COVID-19 virus might be low, but it gets media attention and become the candidate of social media discussion, which might have effects on risk perception, which in turn may determine communities’ behavior in adopting and using these pandemic preventive measures [8]. Understanding the risk perception among people was crucial to understand ways of delivering information for communities using correct information channeling [5, 9]. Few studies conducted previously reveal conflicting findings on the level of perceived risk towards Novel Coronavirus. One survey conducted in Italy identified the effect of age on risk perception and recommend importance of delivering correct information about the disease and its prevention mechanism [10].

The others studies conducted in Iran found moderate risk perception in the community [11, 12]. Opposing to the above two, the study conducted in United States reveals a low level of risk perception [13]. Perceived risk differs across different sociodemographic characteristics including age, educational level, residence, and access to information [9, 11, 14]. The study conducted on COVID-19 risk perception in Vietnam also identified the effect of using social media on risk perception towards COVID-19 [5].

Misinformations that transmit from different media also start helping the effect of this pandemic in affecting people’s behavior towards it. This leads to the development of some popular myths like “COVID-19 doesn’t exist at all”, “it can’t affect people in the hot or cold environment” and “COVID-19 was deliberately created by the people”. These myths have good and bad consequences on health [15]. These bulk of the information which is circulating through multiple channels influences how people think about the disease and their readiness to stick to available preventive methods [5]. This mandate as channeling of basic information should be from trusted sources. Various studies identified the level of utilizing available COVID-19 pandemic preventive techniques and variability of using these methods across different sociodemographic and socioeconomic characteristics [11, 16, 17]. Hence understanding level of risk perception towards the COVID-19 pandemic, identifying myth developed in the communities following this pandemic, and their utilization of available preventive measures has crucial importance in reducing COVID- 19 transmission. Therefore this study was conducted to identify risk perception, community myth, and practice towards COVID-19 pandemic in Southeast Ethiopia.

## Methods

### Study setting, design, and period

A community-based crossectional study was conducted from March to June 2020 among 854 adult populations who were permanent residents of 22 Kebeles in two Zones of the Oromia region, Southeast Ethiopia.

### Inclusion and exclusion criteria

Respondents with an age greater than 18 years were included. Any individual who was not a permanent resident of the study area, critically ill, with hearing impairment, and has changes in consciousness level or cognitive disorders were excluded from the interview.

### Sample size determination

Single population proportion formula for sample size determination was used to calculate the required sample size by taking the proportion of participants with high-risk perception against COVID-19 50%; at 95% confidence level, 5% margin of error, 10% non-respondents, and design effect of 2. This gave the final sample size of 854 individuals.

$$n=\frac{\left(z\propto /2\right)2\;p(1-p)}{d2}$$

After multiplying by design effects = 768. Then 10% of non -respondent rate was added and the overall sample size became 854.

### Sampling procedure and data collection

A multistage sampling technique was used in which woredas and administrative towns were selected after grouping. From selected woredas and administrative towns 22 kebeles (villages) were selected by using lottery methods. Then 854 participants were randomly included from systematically selected households. Data regarding risk perception, community myth, and preventive practice against COVID-19 were collected using tools adapted from previous studies and WHO recommendations [2, 5, 13, 18–24]. Data regarding sociodemographic characteristics and source of information about COVID-19 were collected by using tools adapted from EDHS and previous articles [9, 16, 19, 25]. The questionnaire was initially prepared in English and then translated into Afan Oromo. Translation back to English was done to check for consistency by languages experts. A questionnaire pretest was done before actual data collection on 5% of the sample size. The questionnaire was modified based on pre-test results.

Data were collected by trained data collectors. Two days training was given to the data collector on objectives, relevance of the study, confidentiality, respondent right, informed consent, and on actual data collection procedures. Ethical clearance from Madda Walabu University Research and publication and letter of permission from selected woredas and administrative towns were obtained. After a brief description of the objectives of the study to every study participant oral consent was obtained. Then questionnaires were administered face to face by the data collectors. This is the appropriate approach for people with no formal education. During data collection data collectors gives clarification to the questions misunderstood by respondents. Consistency and completeness of data were checked by investigators every day. After data collection, filled questionnaires were kept carefully.

### Variables measurements

Risk perceptions of respondents were assessed by asking six questions adapted from previously conducted studies [11, 26, 27], Total risk perception score was computed by adding individual responses of these six questions. Then median score was used to categorize the level of risk perception. Respondents those score less than the median were categorized as having low-risk perception, and those scores equal to or above the median were categorized as having high-risk perception regarding the COVID-19 pandemic.

Myths about COVID-19 pandemic were also measured by asking six questions adapted from previous studies [21, 22, 28]. The total myth score was calculated by adding responses to these six questions. The median score was used to label individuals as with wrong myth and not with wrong myth. Accordingly, those scores less than the median were categorized as having no wrong myth, and those scores equal to or greater than the median were categorized as having wrong myth.

Regarding utilization of preventive practice towards COVID-19; respondents were asked twelve questions adapted from World Health Organization advice to the public and previous studies [29–32]. The total practice scores was computed by adding responses to these questions. And median score was used to categorize practice of participants. Accordingly practice of respondents regarding utilization of COVID-19 pandemic preventive measures was categorized as having poor practice and good practice based on their median score computed from these twelve asked questions. Those with a score below the median were categorized as having poor practice and those with a score greater than or equal with median were categorized as having good practice. Refer to appendix one for the questionnaire. The data about sociodemographic variables, access to health care, and source of information were collected by adapting tools from EDHS 2016 and previous studies after some modification.

### Data processing and analysis

Data were checked for completeness and entered to Epidata version 3.1 and were exported to SPSS version 25 for analysis. Data cleaning was done using frequency distribution and descriptive statistics. The scores for risk perception, community myth and practice in utilization of COVID-19 were computed from their respective individual questions responses. Sociodemographic characteristics, access to health care and source of information were summarized using frequency distribution. Average values of all outcomes were calculated and reported. The scores of risk perception, community myth, and preventive practice were compared across different sociodemographic characteristics of respondents. A generalized linear model was used to examine factors associated with risk perception and practice regarding the utilization of available COVID-19 preventive measures. Adjusted odds ratio with a 95% confidence interval was computed and those with a p-value less than 0.05 were reported as significant factors associated with risk perception and practice. Cluster analysis by using a linear mixed model was performed to identify factors associated with community myth. Variables with a p-value less than 0.05 in the linear mixed model were reported as significant factors associated with community myth.

## Results

### Sociodemographic characteristics of respondents

As shown in “Table 1” below 854 respondents participated in this study yielding 100% response rate. Out of the total 854 respondents, 547 (64.1%) were male and 845 (98.9%) were Oromo ethnicity, 611 (71.5%) were rural residents. Regarding occupational status, 499 (58.4%) were farmers, the roles of 660 (77.3%) were father/mother and the majority of them attends primary level of education 335 (39.2%). Concerning their marital status, 645 (75.5%) were married earning the median monthly income of 1675.27 ETB. Around two-thirds of the respondents 534 (62.5%) got information about COVID-19 from TV/radio, 591 (69.2%) of them live near health facility and 265 (30.8%) have a history of substance use mostly khat 228 (26.7%) and 100 (11.7%) of them have a history of chronic illness. Finally, more than two-thirds of the total participants 604 (70.7%) live in their own house.

**Table 1:** Sociodemographic characteristics of study participants.

Variables	Frequency (n)	Proportion (%)
**Gender**		
**Male**	547	64.1
**Female**	307	35.9
**Age in years**		
**Mean**	34.12	
**Standard deviation**	13.29	
**Minimum**	16	
**Maximum**	90	
**Ethnicity**		
**Oromo**	845	98.9
**Amhara**	4	0.5
**Tigre**	3	0.4
**Others**	2	0.2
**Residence**		
**Rural**	611	71.5
**Urban**	243	28.5
**Occupation**		
**Government workers**	113	13.2
**NGO employee**	7	0.8
**Private workers**	161	18.9
**Farmers**	499	58.4
**Others**	74	8.7
**Role in family**		
**Father/Mother**	660	77.3
**Son**	143	16.7
**Daughter**	51	6
**Level of education**		
**No formal education**	213	24.9
**Primary education (1–8)**	335	39.2
**Secondary education (9–12)**	178	20.8
**College and above (12+)**	128	15
**Marital status**		
**Married**	645	75.5
**Single**	183	21.4
**Divorced**	24	2.8
**Others**	2	0.2
**Average monthly income ETB**		
**Median**	1675.27	
**Minimum**	10	
**Maximum**	50000	
**Source of information**		
**Religious leaders**	157	18.4
**TV/radio**	534	62.5
**Social media**	285	33.4
**Health workers**	55	6.4
**Gov’t announcements**	74	8.7
**Another source (like telecom)**	1	0.1
**Distance from health facility**		
**Near health facility**	591	69.2
**Long-distance > 1 hour**	256	30
**Don’t know**	7	0.8
**Substance use**		
**No**	589	69
**Yes**	265	31
**Types of substance used**		
**Khat**	228	26.7
**Cigarettes smoking**	33	3.9
**Alcohol drinking**	28	3.3
**Other substance**	1	0.1
**History of chronic illness**		
**No**	754	88.3
**Yes**	100	11.7
**House ownership**		
**No**	250	29.3
**Yes**	604	70.7

### Distribution of risk perception, community myth and preventive practices towards COVID-19 in communities, 2020 (n = 854)

Risk perception towards COVID-19 was computed from six questions. Its median score is 19 ranging from six to thirty. Those who have risk perception greater than the median score were classified as having high-risk perception. Accordingly around half of the study participants (415 (48.6%) have high-risk perceptions. Risk perception is the same across gender and residence but comparably higher among those who live near health facilities, non-governmental workers, and have a history of chronic illness.

Community myth was assessed by asking six questions. Based on this 428 (50.1%) of the study participants have the wrong myth about the COVID-19 pandemic. The median community myth was higher among females and urban residents. Concerning its distribution across occupation types, it was higher among NGO workers and the lowest among farmers. Also, the lower community myth was scored among less educated and those with a history of substance use. It was also relatively higher among those with a history of chronic illness.

The scores for practice towards utilization of COVID-19 pandemic was computed from 12 questions and the median score was used to categorize participants. Of all participants, 366 (42.9%) of them had low utilization of the stated preventive practice.

There is no gender difference in using practice to prevent COVID-19. Using these preventive practices was higher among urban residents and those near to health facilities but relatively lower among farmers, those without formal education, and substance users. See “Table 2” below.

**Table 2:** Distribution of risk perception, community myth, and preventive practices towards COVID-19.

Variables	Frequency(n)	Percent(%)
**Risk perception**		
**Minimum**	6	
**Maximum**	30	
**Median**	19	
**Myth**		
** Not all people develop a severe condition**		
**No**	511	59.8
**Yes**	343	40.2
**Eating/contacting wild animals could cause COVID-19**		
**No**	368	43.1
**Yes**	486	59.6
**Traditional medicine could prevent/cure COVID-19**		
**No**	682	79.9
**Yes**	171	20
**COVID-19 was deliberately created by people**		
**No**	796	93.2
**Yes**	58	6.8
**Living in a hot/cold environment could prevent COVID-19**		
**No**	763	89.3
**Yes**	91	10.7
**COVID-19 could be transmitted by mosquitoes/housefly**		
**No**	631	73.9
**Yes**	223	26.1
**Total myth score**	1369	
**Minimum myth score**	0	
**Maximum myth score**	6	
**Median myth score**	2	
**Practice**		
**Going to crowded places**		
**No**	429	50.2
**Yes**	425	49.8
**Stay at home**		
**No**	364	42.6
**Yes**	490	57.4
**Reduce consuming outdoor foods**		
**No**	306	35.8
**Yes**	547	64.1
**Avoid handshaking**		
**No**	208	24.4
**Yes**	646	75.6
**Reduce public transportation**		
**No**	386	45.2
**Yes**	468	54.8
**Frequently wash hands**		
**No**	164	19.2
**Yes**	690	80.8
**Pay more attention to personal hygiene**		
**No**	176	20.6
**Yes**	678	79.4
**Use disinfectants**		
**No**	482	56.4
**Yes**	372	43.6
**Use face mask**		
**No**	148	17.3
**Yes**	706	82.7
**Maintain safe social distance**		
**No**	224	26.2
**Yes**	630	73.8
**Practicing respiratory hygiene**		
**No**	150	17.6
**Yes**	704	82.4
**Avoid touching eyes, nose and mouth**		
**No**	296	34.7
**Yes**	558	65.3
**Total (sum) of practice score**	6904	
**Minimum practice score**	0	
**Maximum practice score**	12	
**Median practice score**	9	

### Factors associated with risk perception, community myth and practices towards COVID-19 in communities, 2020 (n = 854)

#### 1. Factors associated with risk perception towards COVID-19 pandemic

Seven variables were found to be eligible for multivariable generalized linear model analysis based the results from bivariate output. These are residence, level of education, distance from the health facility, history of chronic illness, knowledge about COVID-19, myth in the community, and status of utilizing COVID-19 preventive practice.

In the final multivariable generalized linear model three factors (variables) were found to be significantly associated with risk perception towards the COVID-19 pandemic. Accordingly residence, distances from health facility and underlining myths were significantly associated with risk perception. Hence being a rural resident increase the likelihood of having high-risk perception by 2.4 (AOR 2.4 with 95%CI 1.67, 3.43) when compared to urban residents, living near health facility also increase the likelihood of having high-risk perception by 1.48 (AOR 1.48 with 95% CI 1.06, 2.06) when compared with those live far from health facility as well as having wrong myth also increase the likelihood of having risk perception by 1.39 (AOR 1.39 with 95% CI 1.2, 1.9) when compared to those without wrong myth. See “Table 3” below.

**Table 3:** Generalized linear model results of factors associated with risk perception towards COVID-19 pandemic in East Bale and Bale Zone Southeast Ethiopia.

Variables	Beta	95 Confidence Interval of beta		Hypothesis Test		
		Lower	Upper	Wald Chi-Square	Df	P-value
**Residence**						
Rural	0.873	0.512	1.233	22.486	1	0.000*
Urban	0					
**Occupation**						
Gov’t employees	0.102	-0.511	0.716	0.107	1	0.744
NGO employees	0.533	-1.198	2.265	0.364	1	0.546
Private workers	0.086	-0.486	0.658	0.087	1	0.768
Farmers	-0.04	-0.585	0.498	0.025	1	0.875
Daily laborers	0					
**Distance from HF**						
Near	0.393		0.726	5.356	1	0.021*
Far	0	0.060				
**History of chronic illness**						
No	-0.412	-0.848	0.024	3.428	1	0.064
Yes	0					
**Wrong myth**						
No	-0.332	-0.643	-0.022	4.404	1	0.036*
Yes	0					
**Practice**						
Poor	-0.261	-0.561	0.039	2.899	1	0.089
Good	0					
**Knowledge**						
Poor	-0.216	-0.536	0.104	1.750	1	.186
Good	0					

*Significant factors at P-Value of <0.05, 0 = Reference Category, Df = degree of freedom

#### 2. Factors associated with community myth regarding COVID-19 pandemic

Cluster analysis by using linear mixed model was used to identify factors associated with community myth. Accordingly seven variables were selected for final cluster analysis using a linear mixed model based on bivariate analysis results. These were gender, occupation, residence, distance from the health facility, knowledge regarding COVID-19, level of risk perception, and status of practice regarding utilization of COVID-19 preventive techniques.

In the final multivariable model three variables were found to be significantly associated with community myth. Accordingly being NGO employees, knowledge regarding COVID-19 and status of utilization of COVID-19 preventive techniques were significantly associated with community myth. Being an NGO employee positively related to community myth while poor knowledge regarding COVID-19 and poor utilization of available COVID-19 preventive techniques were negatively associated with the average score of community myth after controlling for the effects of other variables in the model. See “Table 4”.

**Table 4:** Linear mixed model results of factors associated with community myth towards COVID-19 pandemic in East Bale and Bale Zone Southeast Ethiopia.

Variables	Estimate	95% Confidence Interval		Df	T-value	P-value
		Lower	Upper			
**Gender**						
Male	-0.053433	-0.210386	0.103520	841	-0.668	0.504
Female	0					
**Occupation**						
Gov’t worker	-0.248985	-0.577316	0.079346	841	-1.488	0.137
NGO worker	1.466748	0.604466	2.329031	841	3.339	0.001*
Private worker	-0.241984	-0.548858	0.064890	841	-1.548	0.122
Farmer	-0.266046	-0.555788	0.023697	841	-1.802	0.072
Daily laborer	0					
**Residence**						
Rural	0.038332	-0.156203	0.232867	841	0.387	0.699
Urban	0					
**Distance from HF**						
Near	0.006825	-0.173489	0.187140	841	0.074	0.941
Far	0					
**Knowledge**						
Poor	-1.174779	-1.332594	-1.016964	841	-14.611	0.000*
Good	0					
**Risk perception**						
Low	-0.109217	-0.261076	0.042643	841	-1.412	0.158
High	0					
**Practice**						
Poor	-.164501	-.326729	-.002272	841	-1.990	
Good	0					.047*

*Significant factors at P-Value of <0.05, 0 = Reference Category, HF = health facility, Df = degree of freedom

#### 3. Factors associated with practice towards utilization of COVID-19 preventive measures

Generalized linear model was used to identify factors associated with practice towards utilization of COVID-19 preventive measures. Nine variables were found to be eligible for multivariable generalized linear model analysis by using results from the bivariate analysis.

These are gender, age, education, distance from the health facility, substance use, knowledge about COVID-19, underlining myth, the existing level of risk perception, and monthly income. After running a multivariable generalized linear model four variables were found as factors significantly associated with practice towards utilization of COVID-19 preventive measures. Accordingly having educational status of college and above increases the likelihood of practicing COVID-19 preventive techniques by 4.25 and 2.16 (AOR 4.25 with 95% CI 2.35, 7.69 and 2.16 with 95% CI 1.30, 3.63) when compared with those with no formal education and primary education respectively. Living near a health facility and having good knowledge regarding COVID-19 pandemic increase the likelihood of having good practice in utilizing preventive techniques by 2.14 and 1.88 (AOR 2.14 with 95% CI 1.50, 3.06 and 1.88 with 95% CI 1.34, 2.64) respectively. The final factor that affects the utilization of COVID-19 preventive techniques was the underlining myth individuals hold regarding this pandemic. Hence having a wrong myth towards COVID-19 pandemic increases likelihood of having good practice by 1.42(AOR 1.42 with 95% CI 1.02, 1.99) when compared with those without the wrong myth. See “Table 5” below.

**Table 5:** Generalized linear model results of factors associated utilization of COVID-19 pandemic preventive measures, East Bale and Bale Zone Southeast Ethiopia.

Variables	Beta value	Beta 95% Confidence Interval		Hypothesis Test		
		Lower	Upper	Wald Chi-Square	Degree of freedom	P-value
**Gender**						
Male	-0.324	-0.667	0.018	3.454	1	0.063
Female	0					
**Residence**						
Rural	0.181	-0.195	0.557	0.888	1	0.346
Urban	0					
**Level of education**						
No formal education	-1.447	-2.037	-0.857	23.094	1	0.000*
Primary education	-0.773	-1.289	-0.257	8.634	1	0.003*
Secondary education	-0.164	-0.731	0.402	0.322	1	0.570
College and above	0					
**Distance from HF**						
Near	0.763	0.408	1.119	17.741	1	0.000*
Far	0					
**Substance use**						
No	0.321	-0.020	0.662	3.412	1	0.065
Yes	0					
**Knowledge**						
Poor	-0.634	-0.972	-0.296	13.500	1	0.000*
Good						
**Risk perception**						
Low	-0.179	-0.489	0.132	1.273	1	0.259
High	0					
**Wrong myth**						
No	-0.354	-0.688	-0.020	4.322	1	0.038*
Yes	0					
**Age in years**	0.003	-0.010	0.016	0.148	1	0.701

*Significant factors at P-Value of <0.05, 0 = Reference Category, HF = health facility

## Discussion

Epidemics and pandemics are unexpected periodic phenomena. They can happen at any time. Peoples face several challenges during such conditions. The effects and impacts of pandemics are multiple. It can affect every aspect of life physically, mentally, and emotionally. Hence in this study, we have investigated risk perception, community myth, and practices towards COVID-19 pandemic and factors associated with them. The risk perception was assessed by giving due attention to emotional and knowledge aspects.

This study found as an around half of or 415 (48.6%) of populations have high-risk perceptions. This finding was the same as the finding from one study conducted in Iran [33] but is higher than the finding from the study conducted in Germany [34] and lower than the finding from the studies conducted in China and Ghana [35, 36]. The disagreement between the current study and studies mentioned could be due to differences in sociodemographic factors like age, residence, educational level and it also might be due to differences in access to information. Also, there is a time difference between when these studies were conducted. The above-mentioned studies were conducted in the early phase of the pandemic. These indicate as level of risk perception in different communities around the world can be different.

This study also found as a rural residence, living near a health facility and having with the wrong myth were significantly and positively associated with risk perception towards the COVID-19 pandemic. This finding conflicts with the studies conducted in Jordan in which urban residence was positively associated with risk perception [37] and it was also not consistent with the other study conducted in China where residence was not significantly associated with risk perception [35, 36]. But this finding was in line with the study conducted in Iran [38].

The reason for the difference could be due to the difference in characteristics of study participants and the speed at which information reach these population based on the status of usage of social media in these different places.

This study also found the prevalence of wrong myths to be 50.1%. This was higher than the finding from a Hospital-based study conducted in Northwest Ethiopia [39]. The reason for disagreement could be the differences in the study setting. Because the current study was community-based while; the study from Northwest Ethiopia was conducted in a health facility which could bring the difference in the finding. The other possible reason could be the time during which these studies were conducted and the difference in the study participants. The study from Northwest Ethiopia was conducted among patients with chronic illness who possibly have regular follow up in the selected Hospital and have a chance to get the right information from health professionals.

This study also identified factors significantly associated with community myths. Accordingly community myth was significantly associated with occupation, knowledge regarding COVID-19, and level of practice regarding utilization of COVID-19 preventive measures. This finding was in line with the finding from the study conducted in South Africa [28] in which knowledge regarding COVID-19 was significantly associated with community myth. But the finding from the current study was not in agreement with the finding from the study conducted in China in which those who have good practice have low myth towards COVID-19 [22]. The reason for the discrepancy could be the difference in sociodemographic characteristics of respondents, the difference in access to information, and the value these communities give for tradition and rumors.

Also, we have found the proportion of people with good practice regarding COVID-19 which is 57.1%. This finding was in line with the other study conducted in Ethiopia [39] where the proportion of poor practice was 47.3%. This was lower than the finding from studies conducted in Nepal and China [22, 24]. The reason for disagreement could be the difference in sociodemographic characteristics of selected study participants as well as it could be due to the difference in receiving correct information and their access to social media. Higher level of education, living near health facility, with good knowledge about the disease and having wrong myth regarding COVID-19 associated with good practice. This finding was in agreement with other studies conducted in China, Pakistan, and Malaysia [21, 22, 39, 40]. But it was different from what was reported in the study conducted in Sudanese [41]. The reason for the difference between these studies and the current study could be due to the difference in the study setting. Because the current study was conducted in the community and the one from Sudan was an online survey which could bring the difference in study participants. This study identified important findings. But it has limitations. Being a crossectional study this study couldn’t identify the direction of association that means whether factors or outcomes come first. It also uses self-report from respondents which could affect the real finding.

## Conclusion

This study is an important step towards a better understanding of risk perception, community myth, and practices regarding COVID-19 pandemics and associated factors. Accordingly, relatively high-risk perception, wrong community myth, and poor practice regarding utilization of COVID-19 preventive techniques were reported. Different factors associated with risk perception, community myth, and practices were identified. These findings could be important input for modeling interventional activities in the community.

## Supporting information

S1 Questionnaire (DOCX)

S1 Data (SAV)

## References

[1] Bavel J.J.V., et al., Using social and behavioural science to support COVID-19 pandemic response. Nature Human Behaviour, 2020. 4: p. 460–471. doi: 10.1038/s41562-020-0884-z 32355299

[2] Naser A.Y.N., et al., Mental health status of the general population, healthcare professionals, and university students during 2019 coronavirus disease outbreak in Jordan: a cross-sectional study. Brain and Behavior, 2020. 10: p. 1–20. 10.1002/brb3.1730 PMC7361060 32578943

[3] Singhal T., A Review of Coronavirus Disease-2019 (COVID-19). Indian Journal of Pediatrics, 2020. 87(4): p. 281–286. doi: 10.1007/s12098-020-03263-6 32166607 PMC7090728

[4] Wolf M.S., et al., Awareness, attitude and action related to COVID-19 among adults with chronic conditions at the onset of the US outbreak. ACP, 2020: p. 1–10. 32271861 10.7326/M20-1239 PMC7151355

[5] Huynh T.L.D., The COVID-19 risk perception: A survey on socioeconomics and media attention. Economics Bulletin, 2020: p. 758–764.

[6] European Centre For Diseases Prevention and Control, *Outbreak of novel coronavirus disease 2019 (COVID-19)*: *increased transmission globally–fifth update*. 2020: Stockholm.

[7] Ibuka Y., et al., The dynamics of risk perceptions and precautionary behavior in response to 2009 (H1N1) pandemic influenza. BMC Infectiuos diseases, 2010. 10(296): p. 1471–2334. doi: 10.1186/1471-2334-10-296 20946662 PMC2964717

[8] Lennart S., Worry and Risk Perception. Risk Analysis, 1998. 18(1): p. 85–93. doi: 10.1111/j.1539-6924.1998.tb00918.x 9523446

[9] Lohiniva A.-L., et al., Understanding coronavirus disease (COVID-19) risk perceptions among the public to enhance risk communication efforts: a practical approach for outbreaks, Finland, February 2020. Euro Surveillance, 2020. 25(13): p. 1560–7917. 10.2807/1560-7917.ES.2020.25.13.2000317 PMC7140598 32265008

[10] Casanova M., et al., How young patients with cancer perceive the COVID-19 (coronavirus) epidemic in Milan, Italy: Is there room for other fears? Pediatric Blood Cancer., 2020. 67: p. 1–5. doi: 10.1002/pbc.28318 32240567

[11] Mohammad H.T., Roham B., and Ramin S., COVID-19 and Iranian Medical Students; A Survey on Their Related-Knowledge, Preventive Behaviors and Risk Perception. ARCHIVES OF IRANIAN MEDICINE, 2020. 23(4): p. 249–254. doi: 10.34172/aim.2020.06 32271598

[12] Samadipour Ezat, Ghardashi Fatemeh, and Aghaei Nahid, Evaluation of Risk Perception of COVID-19 Disease: A Community-Based Participatory Study. Disaster Medicine and Public Health Preparedness,, 2020: p. 1–8. doi: 10.1017/dmp.2020.311 32873355 PMC7642912

[13] McFadden SarahAnn M., et al., Perceptions of the adult US population regarding the novel coronavirus outbreak. Plos One, 2020. 15(4): p. 1–6. doi: 10.1371/journal.pone.0231808 32302370 PMC7164638

[14] Dryhurst S., et al., Risk perceptions of COVID-19 around the world. Journal of risk research, 2020: p. 1–14.

[15] Mian A. and Khan S., Coronavirus: the spread of misinformation. BMC Medicine 2020. 18(89): p. 1–2. doi: 10.1186/s12916-020-01556-3 32188445 PMC7081539

[16] Khosravi M., Perceived Risk of COVID-19 Pandemic: The Role of Public Worry and Trust. Electronic Journal of General Medicine,, 2020. 17(4): p. 2516–3507.

[17] Wise T., et al., Changes in risk perception and self-reported protective behaviour during the first week of the COVID-19 pandemic in the United States. Royal Societ Publishing, 2020. 7: p. 1–13. doi: 10.1098/rsos.200742 33047037 PMC7540790

[18] Huynh T.L.D., The COVID-19 risk perception: A survey on socioeconomics and media attention. Economics Bulletin, 2020. 40(1): p. 758–764.

[19] Ibuka Y., et al., The dynamics of risk perceptions and precautionary behavior in response to 2009 (H1N1) pandemic influenza. BMC Infectiuos diseases 2020: p. 1471–2334. 10.1186/1471-2334-10-296 PMC2964717 20946662

[20] Naser A.Y., et al., Mental health status of the general population, healthcare professionals, and university students during 2019 coronavirus disease outbreak in Jordan: a cross-sectional study. . 2020: p. 1–20. 10.1002/brb3.1730 PMC7361060 32578943

[21] Jelnov, P., *Confronting COVID-19 Myths*: *Morbidity and Mortality*, in *GLO Discussion Paper*, *No*. 516,, 516, Editor. 2020. p. 1–7.

[22] Swapnajeet S., Susanta Kumar Padhy J.I., and Aseem Mehra S.G., Demystifying the myths about COVID-19 infection and its societal importance. Asian Journal of Psychiatry 2020. 54: p. 1–6. 10.1016/j.ajp.2020.102244 PMC7301136 32593121

[23] Dryhurst Sarah, et al., Risk perceptions of COVID-19 around the world. Journal of Risk Research, 2020. 23(7–8): p. 994–1006.

[24] Sarla G.S., COVID 19: Myths and Facts. MAT journal, 2020. 2(2): p. 5–8.

[25] FEDERAL DEMOCRATIC REPUBLIC OF ETHIOPIA, *Demographic and Health Survey*. 2016: Addis Ababa, Ethiopia.

[26] Casanova M., et al., How young patients with cancer perceive the COVID-19 (coronavirus) epidemic in Milan, Italy: Is there room for other fears?.. Pediatr Blood Cancer, 2020: p. 1–5. doi: 10.1002/pbc.28318 32240567

[27] Dryhurst S., et al., Risk perceptions of COVID-19 around the world. Journal of risk research, 2020: p. 1–14.

[28] Schmidt Tenielle, et al., Myths, misconceptions, othering and stigmatizing responses to Covid-19 in South Africa: A rapid qualitative assessment. Plos One, 2020. 15(12). doi: 10.1371/journal.pone.0244420 33351852 PMC7755184

[29] Ferdous Most. Zannatul, et al., Knowledge, attitude, and practice regarding COVID-19 outbreak in Bangladesh: An onlinebased cross-sectional study. Plos One, 2020. 15(10). 10.1371/journal.pone.0239254 PMC7546509 33035219

[30] WHO, *Mental health and psychosocial considerations during the COVID-19 outbreak*. 2020: Geneva.

[31] Wolf M.S., et al., Awareness, attitude and action related to COVID-19 among adults with chronic conditions at the onset of the US outbreak. Annals of Internal Medicine, 2020. 173(2): p. 1–10. 32271861 10.7326/M20-1239 PMC7151355

[32] Wu Y.-C., Chen C.-S., and Chan Y.-J., The outbreak of COVID-19: An overview. J Chin Med Assoc, 2020. 83: p. 217–220. doi: 10.1097/JCMA.0000000000000270 32134861 PMC7153464

[33] Honarvar Behnam, et al., Knowledge, attitudes, risk perceptions, and practices of adults toward COVID-19: a population and field-based study from Iran International Journal of Public Health, 2020. 65: p. 731–739. doi: 10.1007/s00038-020-01406-2 32583009 PMC7311321

[34] Gerhold L., COVID-19: Risk perception and Coping strategies. Results from a survey in Germany. AG, 2020: p. 1–11.

[35] Serwaa Dorcas, et al., Knowledge, risk perception and preparedness towards coronavirus disease-2019 (COVID-19) outbreak among Ghanaians: a quick online cross-sectional survey. Pan African Medical Journal,, 2020. 35(44): p. 1–7. 10.11604/pamj.supp.2020.35.2.22630 PMC7875746 33623569

[36] He Shan, et al., Analysis of Risk Perceptions and Related Factors Concerning COVID‑19 Epidemic in Chongqing, China. Journal of Community Health 2021. 46: p. 278–285. doi: 10.1007/s10900-020-00870-4 32592160 PMC7318903

[37] Karasneh Reema, et al., Media’s effect on shaping knowledge, awareness risk perceptions and communication practices of pandemic COVID-19 among pharmacists. . Research in Social and Administrative Pharmacy,, 2020. 17: p. 1897–1902. doi: 10.1016/j.sapharm.2020.04.027 32340892 PMC7179508

[38] Samadipour Ezat and Ghardash Fatemeh, Factors Influencing Iranians’ Risk Perception of Covid-19. Journal of Military Medicine, 2020. 22(2): p. 122–129.

[39] Akalu Yonas, Ayelign Birhanu, and Molla Meseret Derbew, Knowledge, Attitude and Practice Towards COVID-19 Among Chronic Disease Patients at Addis Zemen Hospital, Northwest Ethiopia. Dove Press Journal infection and Drug Resistance, 2020. 13: p. 1949–1960. doi: 10.2147/IDR.S258736 32612371 PMC7322118

[40] Amgain Kapil, et al., Myths versus Truths regarding the Novel Coronavirus Disease (COVID-2019) Outbreak. Journal of Karnali Academy of Health Sciences, 2020. 3(7): p. 1–6.

[41] Mohamed Amal Abdelrahim Osman, et al., Knowledge, attitude and practice of the Sudanese people towards COVID-19: an online survey. BMC Public Health, 2021. 21(274): p. 1–7. doi: 10.1186/s12889-021-10319-5 33535995 PMC7856621

