# Supplementary material for: Risk perception, community myth, and practices towards COVID-19 pandemic in Southeast Ethiopia: Community based crossectional study
Source: PLoS One. 2022 Oct 3;17(10):e0275331. doi: 10.1371/journal.pone.0275331 (PMC9529088; doi:10.1371/journal.pone.0275331)
Supplement: S1 Questionnaire — (DOCX) [file pone.0275331.s001.docx]

**Appendix I: Questionnaire**

**1. Sociodemographic characteristics Assessment questions**

| **Serial No** | **Questions** | **Options** | **Remark** |
| --- | --- | --- | --- |
| 101 | Gender | 0. Male 1. Female |  |
| 102 | Age in Years | __________ |  |
| 103 | What is your ethnicity? | 1. Oromo 2. Amhara 3. Tigre   99. Others |  |
| 104 | Residence | 0. Rural  1. Urban |  |
| 105 | What is your occupation? | 1. Government employee 2. NGO employee 3. Private works 4. Farmer   99. Others__________ |  |
| 106 | What is your role in the family | 1. Father/Mother  2. son  3. daughter |  |
| 107 | What is the highest educational level that you had attended? | 1. No formal education 2. Primary education (1-8) 3. Secondary school (9-12 4. Collage and above (12+) |  |
| 108 | What is your marital status? | 1. Married 2. Single 3. Divorced   99. Others |  |
| 109 | How much is the average monthly income of your family? | ETB___________ |  |
| 110 | From where you heard about COVID 19 disease | 1. Religious leader 2. Radio/TV 3. Social media 4. Health Worker home to home education 5. Announcement 6. Other (Specify) |  |
| 111 | What distance you have from health Facility? | 1. Near to health facility 2. Long distance walking more than 1 hour 3. I don’t know |  |
| 112 | Did you have history of using the following substance? (multiple answer possible) | 1. khat  2. cigarette smoking  3. alcohol drinking  99. other specify_________ |  |
| 113 | Did you have history of chronic illnesses? | 0. No  1. Yes |  |
| 114 | Did you live in your own house? | 0. No  1. Yes |  |

**2. Knowledge Assessment questions**

| **Serial No** | **Questions** | **Options** | **Remark** |
| --- | --- | --- | --- |
| 201 | The main clinical symptoms of COVID-19 are fever, fatigue, dry cough, and myalgia. | 0. False 1. True, 2. I don’t know |  |
| 202 | Unlike the common cold, stuffy nose, runny nose, and sneezing are less common in persons infected with the COVID-19 virus. | 0. False 1. True, 2. I don’t know |  |
| 203 | Not all persons with COVID-2019 will develop to severe cases except those who are elderly, have chronic illnesses, and are obese are more likely to be severe cases. | 0. False 1. True, 2. I don’t know |  |
| 204 | Eating or contacting wild animals would result in the infection by the COVID-19 virus. | 0. False 1. True, 2. I don’t know |  |
| 205 | Persons with COVID-2019 cannot infect the virus to others when he/she has no symptom. | 0. False 1. True, 2. I don’t know |  |
| 206 | The COVID-19 virus spreads via respiratory droplets of infected individuals. | 0. False 1. True, 2. I don’t know |  |
| 207 | Ordinary residents can wear general medical masks to prevent the infection by the COVID-19 virus | 0. False 1. True, 2. I don’t know |  |
| 208 | It is not necessary for children and young adults to take measures to prevent the infection by the COVID-19 virus. | 0. False 1. True, 2. I don’t know |  |
| 209 | To prevent the infection by COVID-19, individuals should avoid going to crowded places such as bus stations and avoid taking public transportations. | 0. False 1. True, 2. I don’t know |  |
| 210 | Isolation and treatment of people who are infected with the COVID-19 virus are effective ways to reduce the spread of the virus. | 0. False 1. True, 2. I don’t know |  |
| 211 | People who have contact with someone infected with the COVID-19 virus should be immediately isolated in a proper place for 14 days. | 0. False 1. True, 2. I don’t know |  |

**3. Risk perception assessments**

| 301 | How worried are you personally about Coronavirus/COVID-19 at present? | 1. not at all worried  2.not worried  3.Feel nothing  4. Worried  5. very worried’ |  |
| --- | --- | --- | --- |
| 302 | How likely do you think it is that you will be directly and personally affected by/ catching the Coronavirus/COVID-19the in the next 6 months? - | 1. not at all likely  2. not likely  3.feel nothing  4.likely  5. very likely |  |
| 303 | How likely do you think it is that your friends and family in the country you are currently living in will be directly affected by /catching the Coronavirus/ COVID-19the in the next 6 months? - | 1. not at all likely  2. not likely  3.feel nothing  4.likely  5. very likely |  |
| 304 | The Coronavirus/COVID-19 will NOT affect very many people in the country I’m currently living in | 1.strongly disagree  2. disagree  3. neutral  4. agree  5.strongly agree |  |
| 305 | I will probably get sick with the Coronavirus/ COVID-19 | 1.strongly disagree  2. disagree  3. neutral  4. agree  5.strongly agree |  |
| 306 | Getting sick with the coronavirus/ COVID-19 can be serious | 1.strongly disagree  2. disagree  3. neutral  4. agree  5.strongly agree |  |

| **4. Community myth related questions** | | | |
| --- | --- | --- | --- |
| 4.1 | People who get Covid-19 will get very sick/die | 0. No  1. Yes |  |
| 4.2 | It affects only old people young people don’t have to worry | 0. No  1. Yes |  |
| 4.3 | Garlic and other traditional medicine can prevent or cure Covid-19 | 0. No  1. Yes |  |
| 4.4 | Covid-19 can be transmitted by mosquito/housefly | 0. No  1. Yes |  |
| 4.5 | It was deliberately created by people | 0. No  1. Yes |  |
| 4.6 | Living in hot/cold environment can prevent from it | 0. No  1. Yes |  |

**4. Practice Assessment questions**

| Serial No | Questions | Options | Remark |
| --- | --- | --- | --- |
| 401 | In recent days, have you gone to any crowded place like market? | 0. No 1. Yes 2. I don’t remember |  |
| 402 | In order to prevent contracting and spreading COVID-19, did you reduce going out of you home | 0. No 1. Yes 2. I don’t remember |  |
| 403 | In order to prevent contracting and spreading COVID-19, did you reduce consuming outdoor food | 0. No 1. Yes 2. I don’t remember |  |
| 404 | In order to prevent contracting and spreading COVID-19 did you avoid handshaking and hugging | 0. No 1. Yes 2. I don’t remember |  |
| 405 | In order to prevent contracting and spreading COVID-19, did you avoid public transportations | 0. No 1. Yes 2. I don’t remember |  |
| 406 | In order to prevent contracting and spreading COVID-19, did you frequently wash my hands | 0. No 1. Yes 2. I don’t remember |  |
| 407 | In order to prevent contracting and spreading COVID-19, did you use disinfectant and solutions | 0. No 1. Yes 2. I don’t remember |  |
| 408 | In order to prevent contracting and spreading COVID-19, when do you use facial masks? | 0. Never 1. Only in public and crowded places 2. Most of the time 3. Always 4. No opinion |  |
| 409 | Did you maintain safe social distance with people when going outside home | 0. No  1. Yes |  |
| 410 | Did you practice respiratory hygiene | 0. No  1. Yes |  |
| 411 | Did you avoid touching your eyes, nose and mouth | 0. No  1. Yes |  |
