# Supplementary material for: Risk perception, community myth, and practices towards COVID-19 pandemic in Southeast Ethiopia: Community based crossectional study
Source: PLoS One. 2022 Oct 3;17(10):e0275331. doi: 10.1371/journal.pone.0275331 (PMC9529088; doi:10.1371/journal.pone.0275331)
Supplement: Attachment — Submitted filename: PONE-D-21-40289.docx [file pone.0275331.s003.docx]

This study aims to assess risk perception, community myths, and preventive practice towards COVID-19 in Ethiopia, 2020.

A community-based cross-sectional study was conducted among 854 participants selected using a multistage sampling technique.

From 854 respondents included in this study, 428 (50.1%) have a wrong tale about COVID-19, and 366 (42.9%) have poor practice, respectively. Thus, the study lays out high-risk perceptions, false community myths, and relatively low utilization of available methods towards COVID-19 and their associated factors.

1. On time, I evaluate it is good to investigate the awareness level of the country, in informing the international community that has a wrong tale about COVID-19.

2. Please supplement the discussion further to reinforce the direction of improvement for health promotion against problems of COVID-19 in Ethiopia.

See also the following paper:

In Kim, J., Kim, G. & Choi, Y. Effects of air pollution on children from a socioecological perspective. BMC Pediatr 19, 442 (2019). https://doi.org/10.1186/s12887-019-1815-x

Kim, J.I., Kim, G. Effects on inequality in life expectancy from a social ecology perspective. BMC Public Health 18, 243 (2018). https://doi.org/10.1186/s12889-018-5134-1
