# Supplementary material for: Risk perception, community myth, and practices towards COVID-19 pandemic in Southeast Ethiopia: Community based crossectional study
Source: PLoS One. 2022 Oct 3;17(10):e0275331. doi: 10.1371/journal.pone.0275331 (PMC9529088; doi:10.1371/journal.pone.0275331)
Supplement: Attachment — Submitted filename: RESPONSE TO REVIEWERS.docx [file pone.0275331.s004.docx]

RESPONSE TO REVIEWERS

Thank you for reading and commenting our manuscript. We have made changes as per given comments. The responses were provided in the following table.

Thank you again

| **COMMENTS** | **RESPONSES** |
| --- | --- |
| 1. Please ensure that your manuscript meets PLOS ONE's style requirements, including those for file naming. The PLOS ONE style templates can be found at  <https://journals.plos.org/plosone/s/file?id=wjVg/PLOSOne_formatting_sample_main_body.pdf> and  <https://journals.plos.org/plosone/s/file?id=ba62/PLOSOne_formatting_sample_title_authors_affiliations.pdf> | Thank you for your comment. We have revised our manuscript as per the requirements. Thank you again |
| 2. Please review your reference list to ensure that it is complete and correct. | Thank you for your comment. We have revised our references but we couldn’t find problem. Thank you again |
| 3. We suggest you thoroughly copyedit your manuscript for language usage, spelling, and grammar. If you do not know anyone who can help you do this, you may wish to consider employing a professional scientific editing service. | Thank you for your comments. We have thoroughly edit the manuscript to make it clear and informative. We have highlighted bold where changes were made. Thank you again. |
| 4. Thank you for stating the following financial disclosure:  " The funders had no role in study design, data collection and analysis, decision to publish, or preparation of the manuscript."  At this time, please address the following queries:  a) Please clarify the sources of funding (financial or material support) for your study. List the grants or organizations that supported your study, including funding received from your institution.  b) State what role the funders took in the study. If the funders had no role in your study, please state: “The funders had no role in study design, data collection and analysis, decision to publish, or preparation of the manuscript.” c) If any authors received a salary from any of your funders, please state which authors and which funders. d) If you did not receive any funding for this study, please state: “The authors received no specific funding for this work.”  Please include your amended statements within your cover letter; we will change the online submission form on your behalf. | Thank you for your comments. We have made changes accordingly. Thank you agian |
| 5. Thank you for stating the following in the Funding Section of your manuscript:  "This study was funded by Madda Walabu University. The funders had no role in study design, data collection, and analysis, or preparation of the manuscript."  We note that you have provided funding information that is not currently declared in your Funding Statement. However, funding information should not appear in the Acknowledgments section or other areas of your manuscript. We will only publish funding information present in the Funding Statement section of the online submission form.  Please remove any funding-related text from the manuscript and let us know how you would like to update your Funding Statement. Currently, your Funding Statement reads as follows:  " The funders had no role in study design, data collection and analysis, decision to publish, or preparation of the manuscript."  Please include your amended statements within your cover letter; we will change the online submission form on your behalf. | Thank you for your comments. We have incorporated this comments and make modification accordingly. We have have removed the funding information from acknowledgment section |
| 6. In your Data Availability statement, you have not specified where the minimal data set underlying the results described in your manuscript can be found | Thank you for your comments. We have submitted minimal data as supporting information along with this submission  Thank you again |
| 7. Your ethics statement should only appear in the Methods section of your manuscript | Thank you for your comment. We made it accordingly and write ethics statement on only methods section. Thank you again. |
|  |  |
|  |  |
